# Sphingolipid Dysregulation in Type 2 Diabetes and Its Microvascular Complications

**DOI:** 10.3390/ijms27209043

**Published:** 2026-10-12

**Authors:** Ya Wen, Yayun Lin, Yi Liu, Li Zhao

**Affiliations:** Department of Reproductive and Genetics, The First Affiliated Hospital of Kunming Medical University, Kunming 650032, China

**Keywords:** sphingolipid, ceramide, sphingosine-1-phosphate, T2DM, glucolipotoxicity

## Abstract

Sphingolipids arestructural components of cell membranes and bioactive mediators governing cellular metabolism and intercellular communication. Beyond their classical roles in insulin sensitivity and glucose/lipid homeostasis, ceramide, sphingosine-1-phosphate (S1P), sphingomyelin and glycosphingolipids coordinate through lipid rafts, extracellular vesicles, organelle contact sites and circulating carriers to facilitate signaling among pancreas, liver, adipose tissue, vascular endothelium and kidney. This review summarizes literature on how dietary and endogenous sphingolipids contribute to T2DM and its microvascular complications. Preclinical studies, mainly in cultured cells and rodent models, indicate that overnutrition with excess take of saturated and industrial trans fats promotes ceramide accumulation, which impairs AKT/AMPK signaling, enhancing hepatic gluconeogenesis and β-cell dysfunction, whereas S1P exerts protective metabolic and vascular effects. Whether these mechanisms are similarly dominant in humans remains to be established. Chain-length-specific ceramides generated by distinct ceramide synthases (CerS1–6) determine organelle and intercellular responses in experimental models. In human observational studies, ceramide/S1P ratios and selected sphingolipid species have been associated with metabolic risk, but their diagnostic and risk-stratification value requires clinical validation. We further discuss nutritional patterns, antidiabetic and lipid-lowering agents, and emerging nanomedicine or exosome-based strategies that restore sphingolipid balance. Targeting compartment- and chain-length-specific sphingolipid signaling may provide more precise therapeutic opportunities for T2DM and its complications.

## 1. Introduction

Type 2 diabetes mellitus (T2DM) and its microvascular complications are increasing at an unprecedented rate. Data from the International Diabetes Federation 2025 indicate that the number of people aged 20–79 with diabetes has reached 589 million and diabetes accounts for approximately 3.4 million deaths annually. By 2050, the global number of adults with diabetes is projected to rise to 853 million. T2DM is primarily characterized by insulin resistance, hyperglycemia, and dyslipidemia. Poor glycemic control can lead to serious complications such as diabetic nephropathy (DN), diabetic retinopathy (DR), cardiovascular diseases (CVD) and nonalcoholic fatty liver disease (NAFLD) [1]. 58.4% of treated patients worldwide still have poor glycemic control especially in low- and middle-income countries, highlighting an urgent need for novel therapies [2]. Sphingolipids, including ceramides, sphingomyelins, sphingosine, sphingosine-1-phosphate (S1P) and gangliosides, are far less abundant than glycerolipids, accounting for approximately 2–15% of the total cellular lipidome [3]. Sphingolipids are components of lipid rafts in the cell membrane, modulating insulin sensitivity, glucolipid metabolism and organelle homeostasis [4]. Although recent studies have revealed that imbalanced sphingolipid metabolism plays a critical role in the development and progression of T2DM, this has not been adequately recognized in clinical practice, unfortunately. The benefits of diet and exercise on glucolipid metabolism are well established, but their influence on sphingolipid balance has been largely ignored. The most well-established paradigm in sphingolipid metabolism is the “sphingolipid rheostat” theory, which posits that the dynamic balance between pro-apoptotic ceramide and pro-survival S1P determines cell fate and metabolic status [5]. It has now been found that beyond ceramide and S1P, other sphingolipids and their related enzymes also take part in the sphingolipid rheostat. How do key sphingolipid enzymes regulate insulin signaling and lipid homeostasis? These questions warrant systematic exploration. We propose that chain length and compartment-specific sphingolipid signaling, rather than total sphingolipid abundance, determines metabolic outcomes in T2DM. This review critically evaluates the evidence for this concept across dietary, mechanistic and biomarker. Emerging therapeutic strategies such as lipid nanoparticles targeting sphingolipid-metabolizing enzymes, modulation or exosome-coupled S1P have shown potential in diabetes. These are not just laboratory curiosities. They could offer significant scientific value and translational potential for clinical practice.

## 2. Materials and Methods

This article was a narrative review. The search strategy was developed by combining sphingolipid biology and diabetes pathophysiology. For sphingolipids, we included terms for major metabolites (ceramide, sphingosine, S1P), related enzymes (acid and neutral sphingomyelinase, ceramidase, sphingosine kinase 1/2, S1P phosphatase, S1P lyase), and functional descriptors (sphingolipid rheostat, ceramide/S1P ratio). For diabetes, we used terms covering type 2 diabetes and its pathophysiological features (insulin resistance, insulin signaling, glucose uptake, lipotoxicity, pancreatic islet function), as well as its complications (microvascular like DN and DRdisease). Gestational diabetes was included only when it formed T2DM risk. Type 1 diabetes was not systematically searched and is mentioned only as comparative background where relevant. The search was limited to human studies, animal models and in vitro studies published in English, with no restriction on sample size but requiring full-text availability. The final search was performed in September 2026. Two reviewers independently screened titles and abstracts; discrepancies were resolved by consensus or by a third reviewer. The figures were generated using PicDoc, an AI-based tool, and were also revised by the authors.

## 3. Sphingolipid Biosynthesis and Related Glucolipotoxicity

**Figure 1:**
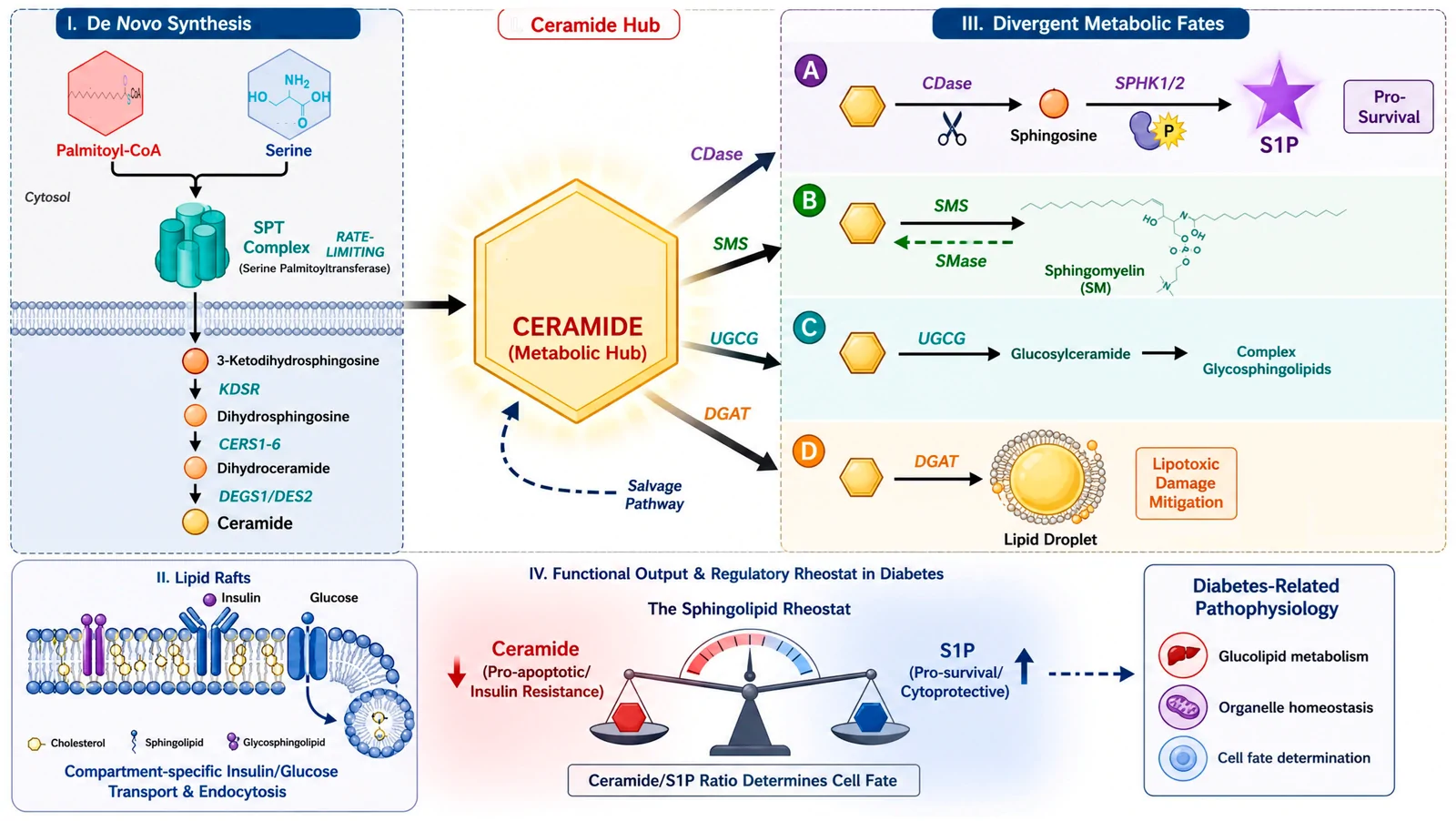
Ceramide is a central hub in sphingolipid metabolim in diabetes. Ceramide accumulation branches into three major pathways, including:de novo synthesis, the sphingomyelinase pathway, and the salvage pathway. The most well-established paradigm in sphingolipid metabolism is the “sphingolipid rheostat” theory, positing that the dynamic balance between pro-apoptotic ceramide and pro-survival S1P which determines cell fate and metabolic status. (Letters A–D indicate divergent ceramide metabolic fates. Solid arrows indicate enzymatic conversion; dashed arrows indicate salvage/reverse pathways. In the rheostat, red = ceramide (pro-apoptotic/insulin resistance), blue = S1P (pro-survival/cytoprotective); ↓/↑ indicate decrease/increase).

Ceramide is central to sphingolipid metabolism, which branches into three major pathways. The de novo route starts with the serine palmitoyltransferase (SPT) complex, which condenses palmitoyl-CoA and L-serine to generate 3-ketodihydrosphingosine. This intermediate is subsequently metabolized by 3-ketodihydrosphingosine reductase (KDSR) to yield dihydrosphingosine (DHS). Six distinct ceramide synthases (CerS1-6) acylate DHS to produce dihydroceramide (dhCer), which is finally desaturated by sphingolipid Δ4-desaturase (DEGS1/2) through the trans-double bond to form ceramide. The second pathway involves sphingomyelinase (SMase), which degrades sphingomyelin (SM) back to ceramide. The sphingomyelin phosphodiesterase 1 (*SMPD1*) gene encodes the protein known as acid sphingomyelinase (ASM). In addition, there are neutral sphingomyelinase (nSMase) and alkaline sphingomyelinase (alk-SMase). The third is the salvage pathway. It recycles sphingosine to ceramide, maintaining intracellular sphingolipid turnover. Ceramide also serves as the precursor for multiple bioactive lipids. It can be deacylated by ceramidase (CDase) to release sphingosine (SPH) and free fatty acids (FFAs), which are further phosphorylated to sphingosine-1-phosphate (S1P) by two rate-limiting kinases, sphingosine kinase 1 (SPHK1) and sphingosine kinase 2 (SPHK2). Alternatively, it can be glycosylated by UDP-glucose ceramide glucosyltransferase (UGCG) to form glucosylceramide (GlcCer), or it can receive a phosphocholine head group from sphingomyelin synthases (SMS) to produce SM [6,7]. Based on the above regulatory framework, this review further explores how the pathological roles of dysregulated sphingolipid metabolism contribute to the pathology of T2DM, diagnosis and stratification, and targeting intervention strategies (Figure 1).

Glucolipotoxicity is a pathological state characterized by the deleterious effects of both hyperglycemia and hyperlipidemia on various cell types, triggering oxidative stress, mitochondrial injury, endoplasmic reticulum (ER) stress and inflammation, which are detrimental to pancreatic β-cells, as well as to metabolic organs including the liver, skeletal muscle, and kidneys [8,9]. Sphingolipids exert their effects largely through interaction with key signaling proteins such as protein kinase B (AKT), AMP-activated protein kinase (AMPK), and mammalian target of rapamycin (mTOR) [10,11]. Palmitate promotes the intracellular accumulation of ceramide and sphingosine, activating PP2A, a serine/threonine phosphatase, which in turn dampens insulin-stimulated phosphorylation of AKT/PKB and glycogen synthase kinase-3 beta (GSK-3β), thereby blunting glycogen synthesis and insulin signaling. In line with this, okadaic acid, a protein PP2A inhibitor, was shown to block ceramide-induced hepatic glucose production [12]. Ceramides also promoted glucagon-stimulated hepatic gluconeogenesis by dephosphorylation of cAMP-responsive element-binding protein (CREB)-regulated transcription coactivator 2 (CRTC2) at Ser171. This modification drived CRTC2 nuclear translocation, activated CREB, and induced gluconeogenic genes such as glucose-6-phosphatase (G6Pase) and phosphoenolpyruvate carboxykinase (PEPCK) [13]. Inr the de novo pathway of ceramide, liver-specific *Sptlc2* transgenic mice exhibit elevated fasting blood glucose, along with inhibited phosphorylation of insulin receptor beta (IRβ). Moreover, the transcriptional activation of *Sptlc2* appears to be mediated by spliced X-box binding protein 1 (sXBP1) in response to ER stress [14]. SPTLC3-derived sphingomyelins containing d16 sphingoid bases are localized to the plasma membrane where they sustain normal adenylate cyclase activity. This enables glucagon-stimulated cAMP/CREB signaling, which drives transcription of genes involved in gluconeogenesis and glucose transport (*PCK1*, *G6PC*, *GLUT2*) and raises fasting blood glucose [10]. *Degs1* knockout in mice significantly improves glucose tolerance, suggesting that ceramides, but not dhCer, which lacks the critical 4,5-trans double bond, are required for suppressing AKT signaling and impairing glucose utilization [15]. Conversely, in preadipocytes, the ablation of *Degs1* increases the dhCer/ceramide ratio, suppressing PPARγ-induced adipogenesis and lipogenesis. Consequently, oxidative stress, inflammation, and apoptosis were exacerbated [16]. ASM, a key enzyme in sphingomyelin catabolism, is directly linked to diabetes. When researchers injected this enzyme into lean mice and obese (ob/ob) mice, ceramide levels in the liver went up, and insulin resistance got worse [17]. In diabetic rats, lowering ASM increased glucose uptake and the expression of glucose transporter 4 (GLUT4) in vascular endothelial cells. It also reduced ceramide production and improved insulin signaling by altering the phosphorylation levels of IRS-1 at Ser307 and AKT at Ser473 [18]. In β-cells, inflammatory stress triggers the production of ceramide-rich extracellular vesicles (EVs) through nSMase2. These EVs act as paracrine effectors and suppress the glucose-stimulated insulin secretion (GSIS) peak in healthy β-cells [19]. For the degradation of ceramide, CDase overexpression in cultured C2C12 myotubes relieves the suppression of AKT/PKB activation, glucose uptake, and anabolic metabolism imposed by ceramide and long-chain saturated FFAs [20]. Collectively, these findings suggest that ceramide, rather than sphingosine, serves as the key regulatory molecule driving insulin signaling impairment and ectopic lipid accumulation in cultured cells and mouse models. Whether ceramide plays an equally dominant causal role in human insulin resistance remains to be determined.

## 4. Sphingolipids from Dietary Sources and Systemic Outcomes

**Figure 2:**
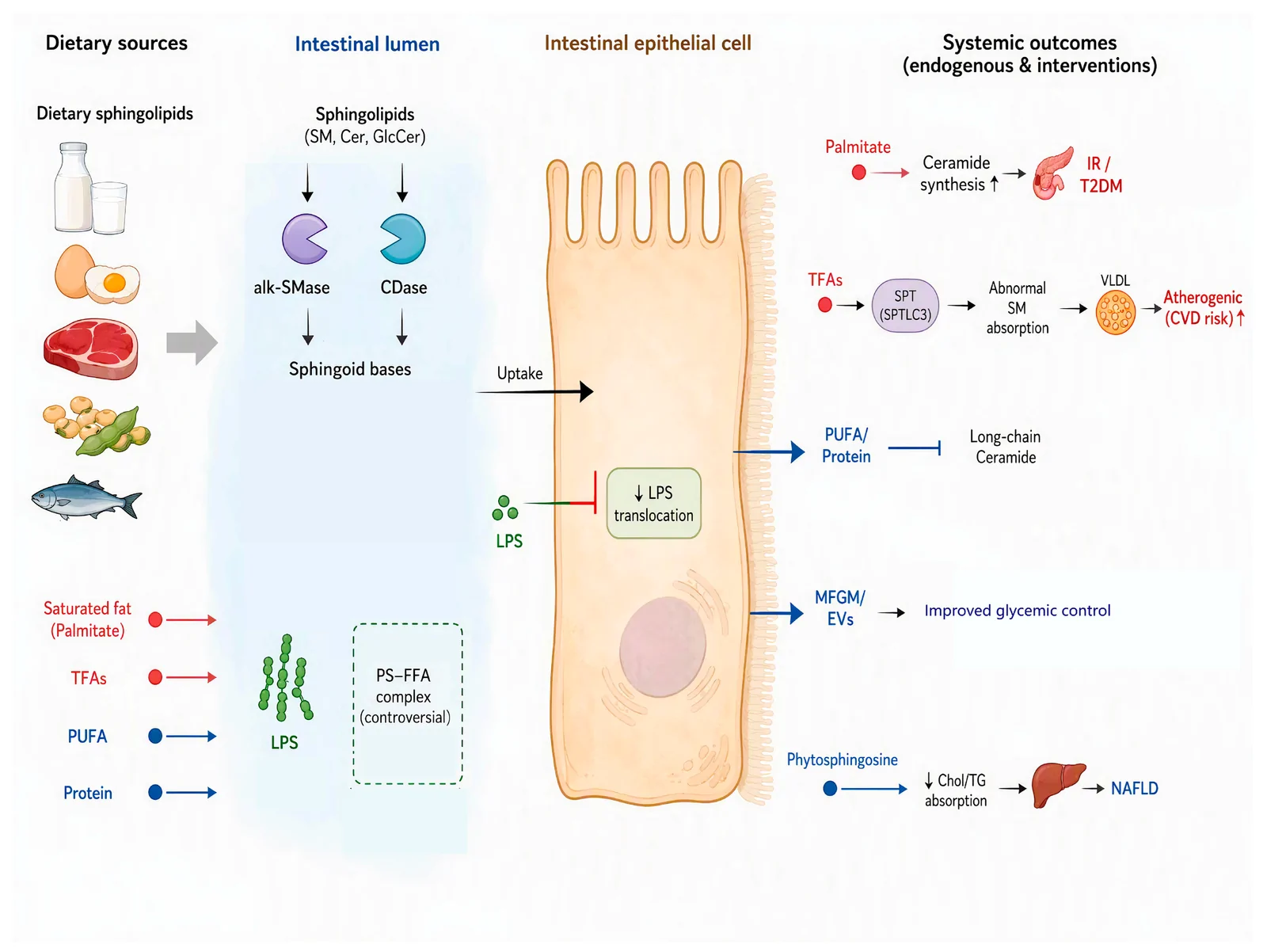
Sphingolipids from dietary sources and their systemic outcomes. Overnutrition and Western-style diets rich in saturated fatty acids and industrial trans fats promoteceramide and abnormal SM accumulation, contributing to IR/T2DM and atherogenic CVD risk. Conversely, diets enriched in unsaturated fatty acids and protein-rich regimens effectively are associated with reduced long-chain ceramide levels, potentially counteracting ceramide accumulation. The FFA-PS complexes may inhibit FFA uptake, whereas cholesterol absorption may be reduced through distinct physicochemical mechanisms, such as altered mixed micelle solubilization. The dashed box indicates that the mixed micelle hypothesis remains controversial. (Red = saturated fat/palmitate/TFAs; blue = PUFA/protein/phytosphingosine. Solid arrows = promotion/flow; T-bars = inhibition; dashed box = controversial mechanism).

Sphingolipids originate from both endogenous de novo synthesis and dietary intake. As the most abundant dietary sphingolipid, SM is predominantly found in animal-derived foods (e.g., milk, eggs, meat) and certain plant-based sources, such as soybeans, while its content is low in fruit and vegetables [21,22,23]. Aquatic products are abundant in diverse sphingolipids, including SM, cerebrosides, gangliosides and deoxysphingolipids. Dietary sphingolipid content is tightly modulated by both caloric intake and cooking methods [24]. Japanese dietary studies demonstrate that low-calorie diets (1600–1700 kcal/day) provide 128 mg cerebrosides and 45 mg sphingomyelins, whereas high-calorie diets (3000 kcal/day) supply 292 mg cerebrosides and 81 mg sphingomyelins from cereals, vegetables, legumes and fruits [25]. Once ingested, dietary sphingolipids are hydrolyzed by intestinal alk-SMase and CDase to produce absorbable ceramide and sphingoid bases. These products are then taken up by intestinal epithelial cells for sphingolipid recycling. At appropriate doses, dietary sphingolipid supplementation helps improve dyslipidemia and NAFLD, largely by reducing the intestinal absorption of cholesterol, triglycerides and FFAs. They also help support the gut-liver axis by limiting the translocation of lipopolysaccharide (LPS) and dampening LPS-mediated pro-inflammatory signals [23]. In addition, dietary fatty acid composition profoundly affects circulating sphingolipid profiles. Higher saturated fat intake is positively correlated with elevated plasma levels of sphingolipids containing a 16:1:O_2_ backbone, including ceramides, monohexosylceramides, dihexosylceramides, SM, and sphingoid base phosphate (SPBP). In contrast, high polyunsaturated fat intake reduces the concentration of long-chain ceramides and long-chain hexosylceramides. Protein intake, interestingly, shows an inverse relationship with most sphingolipid subclasses, except for sphingolipids containing d16:1 sphingoid base [26]. Overall, a diet high in saturated fat but low in polyunsaturated fat or protein is related to the accumulation of sphingolipids which are closely related to the pathogenesis of T2DM and CVD. Palmitate, the most abundant saturated fatty acid in Western-style diets, acts not only as a classical metabolic substrate but also as a potent lipotoxic mediator. As a direct precursor for de novo ceramide synthesis, excess palmitate fuels the development of insulin resistance (IR) and T2DM. Industrial trans fatty acids (TFAs), owing to their straight-chain structure, are incorporated into the sphingolipid biosynthetic pathway several-fold more efficiently than cis fatty acids under catalysis of SPT (particularly the SPTLC3 subunit), leading to the generation of structurally aberrant SM species. These sphingolipids are efficiently packaged into VLDL and secreted into the circulation, constituting an atherogenic pathway independent of cholesterol metabolism [27]. In animal models and small human intervention studies, abundant milk-derived fat globule membranes (MFGMs) and EVs are characterized by unique very-long-odd-chain ceramide and SM. They also reduce cardiometabolic risk-related lipid biomarkers, thereby exerting systemic lipid-lowering effects and improving glycemic control [28]. A 16-week dietary intervention with MFGMs and EVs markedly raised very-long-odd-chain SM levels and accelerated overall lipid metabolic flux in mice [29]. These findings suggest that milk-derived sphingolipids may have favorable metabolic effects. However, the precise effects of dietary sphingolipids on glycemic control and T2DM outcomes in humans remain poorly understood. Further human intervention studies are required. Beyond their effects on glycemic control, dietary sphingolipids also modulate systemic lipid metabolism. In APOE*3-Leiden mice, dietary sphingolipid supplementation reduced plasma cholesterol and triacylglycerol levels in a dose-dependent manner. 1% phytosphingosine (PS) supplementation decreased plasma levels of cholesterol and triacylglycerol by 57% and 58%, respectively, thereby attenuating NAFLD progression. These systemic lipid-lowering effects are likely to originate, at least in part, from reduced intestinal lipid absorption. Consistent with this possibility, PS is considered to inhibit intestinal absorption of dietary cholesterol (50%) and FFAs (40%) through distinct mechanisms [30]. For FFAs, ionic interactions between the carboxylate groups of FFAs and the protonated primary amine of PS may form FFA-PS complexes that block intestinal uptake. In contrast, cholesterol is a neutral molecule and cannot participate in ionic bonding. Its absorption may instead be reduced through physical interactions with PS or related sphingoid bases, such as the formation of condensed lipid complexes with cholesterol, or through altered cholesterol solubilization in bile salt mixed micelles. Whether PS directly interacts with cholesterol at the enterocyte membrane or indirectly alters micellar cholesterol availability remains to be determined. These proposed mechanisms are based largely on preclinical models and physicochemical studies, and their relevance to human intestinal cholesterol absorption requires further investigation (Figure 2).

## 5. N-Acyl Chain and Sphingoid Base Specificity of Sphingolipids in T2DM and Its Complications

Sphingolipid structural diversity is determined by two independent structural dimensions: the sphingoid base (e.g., d16:1, d18:1, d18:0, or t18:0) and the N-acyl chain (e.g., C16:0, C18:0, or C24:0). Throughout this review, we specify whether chain length refers to the sphingoid base or the N-acyl chain. Ceramides have context-dependent cytotoxic and metabolic effects that depend on the sphingoid base, the N-acyl chain length and saturation, and subcellular localization [31]. Clinical studies have found that serum C20–C24 N-acyl ceramides are positively associated with several metabolic risk markers such as body weight, fat mass, BMI, fasting blood glucose, as well as serum triglycerides, total cholesterol, and low-density lipoprotein cholesterol (LDL-C). Additionally, ceramides with C16:0, C18:0, C20:0, and C22:0 N-acyl chains have been shown to be positively associated with an increased risk of hyperglycemia, an effect primarily attributed to the de novo biosynthesis of ceramide in the liver [13]. CerS1-6 catalyze the N-acylation of sphingoid bases. This step also plays a key role in determining the acyl-chain length. CerS1 mainly produces C18:0 N-acyl ceramides. CerS2 primarily generates very-long-chain C20:0-C26:0 N-acyl ceramides. CerS3 generates very-long-chain ≥C26:0 N-acyl ceramides. CerS4 mainly produces C18:0 to C20:0 N-acyl ceramides. CerS5 and CerS6 mainly produce C14:0 and C16:0 N-acyl ceramides [32]. Tissue-specific dysregulation of CerS2 induces distinct metabolic and organ-specific pathologies. Cardiomyocyte-specific overexpression of *Cers2* triggers mitochondrial dysfunction, oxidative stress, and cardiomyocyte apoptosis. Promoting CerS2 phosphorylation in white adipose tissue enhances the biosynthesis of C20–C24 N-acyl ceramides, which suppress GDF15 expression, ultimately driving excessive food intake and metabolic dysfunction [33]. Whereas β-cell-specific ablation of *Cers2* selectively reduces insulin synthesis and secretion, leading to glucose intolerance. It has been considered that C20–C24 ceramides may disrupt their interaction with ER-Golgi transport proteins, thereby inhibiting PCSK1-mediated proinsulin processing and maturation [34]. In cultured podocytes and mouse models, CerS6-derived Cer(d18:1/16:0) is predominantly elevated in podocytes relative to tubular cells in diabetic nephropathy, and this lipid is negatively correlated with estimated glomerular filtration rate (eGFR) while showing positive correlations with serum creatinine and proteinuria. CerS6-derived Cer(d18:1/16:0) can directly bind to the mitochondrial channel protein VDAC1 at the Glu59 residue, triggering mitochondrial DNA (mtDNA) release into the cytosol and activating the cGAS-STING pathway, ultimately promoting renal inflammatory injury [35]. Whether this pathway is similarly activated in human diabetic kidneys requires further validation. In a mouse model of type 1 diabetes, the accumulation of D-serine and the depletion of L-serine in the skin inhibit SPT activity and ceramide output. Concurrently, ASM is activated and elevates the production of proinflammatory lipid mediator ceramide-1-phosphate (C1P). These mediators downregulate *Cers3* expression and long-chain ceramide synthesis while upregulating *Cers5* and *Cers6* expression, which generate shorter-chain ceramides. This imbalance in ceramide chain lengths compromises the structural integrity of stratum corneum lamellae and may contribute to the pathophysiology of diabetic ulcers. These findings are specific to type 1 diabetes and may not be directly generalizable to T2DM [36]. Beyond the above pathogenic roles, ceramide chain-length ratios have been associated with cardiometabolic risk in human observational studies and may serve as candidate biomarkers. An elevated molar ratio of C16:0 N-acyl to C24:0 N-acyl ceramides has been associated with cardiometabolic morbidity [37], but prospective validation and analytical standardization are needed before clinical use. Accordingly, if validated in clinical studies, targeting specific chain-length pools, rather than global sphingolipid synthesis, may represent a more precise and effective therapeutic strategy for T2DM and its complications.

## 6. Sphingolipid Dysregulation in CVD and Microvascular Complications

Sphingolipids exert widespread systemic pathological effects and serve as key drivers of both CVD and microvascular complications. In the heart, ceramide enriched in the outer mitochondrial membrane increases cytochrome c permeability and induces the mitochondrial apoptotic pathway. Atypical d16-sphingolipids can directly bind to the mitochondrial Complex I (CI). Cardiomyocyte-specific knockout of SPTLC3 diminishes mitochondrial integrity and CI activity, thereby lowering oxidative stress and improves post-ischemic survival, which preserves cardiac function [38]. Beyond the heart, the vasculature is also profoundly affected by sphingolipid signaling. Nitric oxide (NO) modulates sphingolipid-mediated cellular behaviors, particularly those involving ceramide and S1P which govern endothelial cell apoptosis, proliferation, and differentiation [39,40]. In vascular endothelial cells, ceramide activates PP2A and inhibits endothelial nitric oxide synthase (eNOS) activity, leading to endothelial dysfunction and hypertension. Moreover, ceramide analogs stimulate ROS production by activating NADPH oxidase and disrupting the mitochondrial electron transport chain. Intriguingly, although ceramides induce superoxide anion generation, co-treatment with superoxide scavengers does not attenuate ceramide-mediated eNOS inhibition, indicating the existence of ROS-independent regulatory mechanisms [41,42]. Inflammatory cytokines such as TNF-α further aggravate vascular injury by activating ASM and promoting de novo ceramide synthesis. Acute, flow-induced ceramide generation may be beneficial and can be converted to S1P to support endothelial NO synthesis. Work reported by Julie K. Freed and colleagues showed that acutely generated ceramides produced by ASM during blood flow exert beneficial effects on flow-induced arteriolar dilation, and these newly synthesized ceramides can be further metabolized into S1P to drive endothelial NO synthesis [43]. Under acute stress, endogenous catecholamines suppress ceramide synthesis to relieve its inhibitory effect on lipolysis and facilitate energy mobilization. However, chronic lipid overload and β-adrenergic desensitization abrogate this protective mechanism, leading to toxic ceramide accumulation, insulin resistance, and hepatic steatosis. This temporal distinction is critical when interpreting therapeutic strategies aimed at global ceramide suppression. Extending to the retina, sphingolipid metabolism is markedly correlated with retinal function in DR. In a human vitreous study of patients with proliferative DR (PDR), the ratio of vitreous C16:0 N-acyl ceramides to very-long-chain ≥C26 N-acyl ceramides was increased [44]. In cultured retinal endothelial cells and animal models, alkaline ceramidase 2 (ACER2) reduces ceramide content, and its overexpression mitigates endothelial barrier disruption, whereas ACER2 silencing exacerbates retinal vascular injury [45]. Whether similar changes occur in NPDR remains unknown. Whether the vitreous ceramide ratios change before the onset of PDR to guide early intervention requires prospective studies in NPDR cohorts. In the kidney, excess dietary or circulating palmitate undergoes aberrant glomerular filtration and is internalized via apical fatty acid transport protein 2 (FATP2) alongside constitutive basolateral uptake. This overload exceeds the buffering capacity of lipid droplets and diverts palmitate flux toward the ER to synthesize C16:0 ceramide. The newly generated ceramides integrate into ER membranes and induce a gel-like phase transition, decreasing membrane fluidity. Such stress subsequently activates PERK and IRE1, leading to ER fragmentation, cellular damage, and progressive deterioration of renal function [46]. Current research mainly focuses on ceramides. More studies are needed to elucidate the pathogenic roles of other sphingolipid metabolites in target organ injury.

## 7. Sphingolipids as Candidate Biomarkers for Disease Risk and Patient Stratification

Circulating sphingolipids have been associated with metabolic disease risk in several human observational studies and are candidate biomarkers for risk stratification. However, few have been clinically validated for diagnosis, prognosis, or risk prediction. In a cross-sectional analysis from the MIDUS cohort, ceramides with C18:0 and C22:0 N-acyl chains were positively associated with the prevalence of T2DM, whereas three lactosylceramides (LacCer(d18:1/14:0), LacCer(d18:1/16:0), and LacCer(d18:1/24:1)) were inversely associated with insulin resistance and T2DM prevalence. Because these are cross-sectional data, they cannot establish prediction of disease onset. Longitudinal data from the Spanish PREDIMED cohort further confirmed that a reduced LacCer/ceramide ratio was associated with higher T2DM incidence [47]. These studies showed associations between plasma sphingolipids and T2DM risk, but they do not establish causality or intracellular metabolic flux. It has been proposed, mainly from preclinical models, that ceramides are glycosylated to GlcCer, which may maintain insulin signaling via remodeling of lipid rafts in early T2DM; sustained glucolipotoxicity may impair this compensation and decrease LacCer/ceramide accumulation. Plasma concentrations are not a substitute for intracellular metabolic flux, and this hypothesis requires direct validation. In the prospective EPIC-Potsdam study, a nested case–cohort analysis revealed that C18:0 and C22:0 N-acyl ceramides, along with dhCer C20:0 and C22:2, were positively associated with T2DM risk, whereas dhCer C26:1 was inversely linked. For cardiovascular outcomes, C16:0 N-acyl ceramides and dhCer C20:0 and C22:2 were associated with higher incidence of CVD. Importantly, disrupted homeostasis of ceramides and dhCer was closely tied to the excessive red meat intake and partially mediated the protective association between coffee consumption and lower T2DM risk [48]. Sphingolipids, including reduced phospholipids and SM, also play a role in obesity phenotyping [49]. Cer(d18:1/24:1) has been reported to differentiate metabolically healthy from metabolically unhealthy obesity. However, whether it can be used for precise clinical risk stratification requires validation in independent prospective studies. In patients with T2DM, hepatic fat content is positively correlated with specific ceramides: Cer(d18:1/18:0), Cer(d18:1/22:0), and Cer(d18:1/24:0), independent of conventional NAFLD risk factors [50]. Dysregulated sphingolipid biosynthesis has been associated with both prevalent and incident of T2DM, and the *CERS2* gene variant rs267738 has been implicated. In postpartum women with a history of gestational diabetes mellitus (GDM), a condition that predisposes to T2DM, the CERS2 variant rs267738 was associated with T2DM risk. Mice with whole-body *Cers2* knockout or targeted rs267738 allele knock-in exhibit glucose intolerance and impaired insulin secretion due to pancreatic β-cell dysfunction [51]. The *SPTLC3* variant rs680379 is associated with lower *SPTLC3* expression. Individuals carrying the *SPTLC3* rs680379 GG genotype have a significantly lower risk of T2DM than those with the A allele (GA or AA). This polymorphism is significantly associated with circulating levels of ceramides (Cer(d18:1/22:0) and Cer(d18:1/24:0)) [52,53]. Data from the FINRISK 2002 and WECAC studies consistently demonstrated that the Cer(d18:1/18:0)/Cer(d18:1/16:0) ratio was associated with T2DM onset, and this ratio decreased following a weight loss of 5% or more [54]. These observational findings require validation before the ratio can be considered as a clinically useful predictive biomarker. Turning to cardiovascular outcomes, the elevated ratio of C16:0 to C24:0 N-acyl ceramides is positively associated with coronary microvascular resistance, highlighting the critical role of Cer(d18:1/16:0) in diabetic vascular dysfunction and atherosclerosis [55,56]. Cellular evidence shows that ceramides induce cellular senescence, while S1P exerts cytoprotective effects [57]. The sphingolipid rheostat has been proposed as a potential biomarker of DM and its microvascular complications, but its clinical utility remains to be validated. The accumulation of ceramide inhibits subsequent AKT signaling and hepatic gluconeogenesis, whereas S1P exerts protective effects by activating the AKT/AMPK signaling pathway. The imbalance between these two signaling molecules is critically involved in the pathogenesis of T2DM [58]. However, the transition of these biomarkers into routine clinical practice requires standardization of analytical platforms and further validation in diverse populations and prospective intervention trials.

## 8. Pharmacological Modulation of Sphingolipid Metabolism

Clinical antidiabetic drugs exert metabolic protective effects partly through modulation of sphingolipid homeostasis. For instance, the insulin sensitizer rosiglitazone significantly increases plasma S1P levels and improves systemic glucose homeostasis [59]. Sodium-glucose cotransporter 2 inhibitors (SGLT2i) remodel the overall lipid profile, encompassing lysophosphatidylcholine (LPC), phosphatidylcholine (PC), phosphatidylethanolamine (PE), SM, triacylglycerol (TG) and ceramides [60]. In diabetic patients treated with SGLT2i, serum Cer(d18:0/16:0), Cer(d18:0/22:0) and Cer(d18:0/24:1) levels were markedly reduced [61]. Beyond antidiabetic agents, lipid-modulating drugs also regulate sphingolipid levels. Statins (simvastatin, rosuvastatin) reduced plasma ceramide concentrations by 20–30% relative to ezetimibe or placebo. Likewise, treatment with a PCSK9 inhibitor for 2–12 months decreases circulating ceramide levels by approximately 30%, accompanied by significant LDL-C reduction [32]. In addition to pharmacological interventions, lifestyle modifications exert profound effects on sphingolipid metabolism. ASM is significantly upregulated after high-intensity interval training (HIIT) in patients with T2DM. HIIT selectively increases specific ceramide species (C16:0, C20:0, C22:0, C24:0, C24:1 N-acyl ceramides) and primarily facilitates exercise-induced mitochondrial quality control rather than promoting lipotoxicity [62]. Emerging therapeutic approaches aimed at reducing elevated ceramide levels are also of great therapeutic value. Hepatocyte-specific *Sptlc2* silencing via LNP-formulated siRNA lowers hepatic ceramide levels and hepatic steatosis through inhibition of CD36-dependent fatty acid uptake and SREBP-1c-mediated de novo lipogenesis [63]. Inducible *Degs1* knockout in mice protects mice from glucose intolerance, insulin resistance, and hepatic steatosis. Meanwhile, myriocin (0.3 mg/kg/day), an inhibitor of serine palmitoyltransferase (SPT) was administered to Otsuka Long-Evans Tokushima Fatty rats. It modestly blunted the increases in body weight, fasting plasma glucose, FFAs, and circulating insulin levels. It also alleviated albuminuria and reduced ceramide concentrations within the kidney cortex [64].

Beyond these direct enzyme inhibitors, adipocyte-derived sphingolipids function as critical nutrient-sensitive signaling molecules, orchestrating systemic adaptation in energy expenditure and whole-body metabolic homeostasis [65]. The adiponectin receptor agonist AdipoRon ameliorates cardiac insulin resistance, inflammation, and lipotoxicity in db/db mice. Mechanistically, AdipoRon upregulates AdipoR1 and AdipoR2 via the p-AMPK/FOXO1-induced AKT phosphorylation pathway resulting from PP2A inhibition. It enhances CDase function, thereby reducing cardiac ceramide levels and increasing S1P production in cardiomyocytes [66]. Furthermore, adipokine IL-11/IL-11Rα signaling acts as a negative regulator of thermogenesis. Adipocyte-specific *IL-11Rα*-knockout mice exhibit elevated whole-body energy expenditure and improved glucolipid metabolism under HFD conditions through upregulation of sphingosine kinase 1 (*Sphk1*) expression and S1P production, which remodels intracellular calcium cycling from ER to mitochondria in beige adipocytes [67]. S1P signaling dysfunction also contributes to diabetic platelet abnormalities. Platelets from T2DM exhibit reduced aggregation ability, impaired S1P responsiveness and downregulated S1PR2 expression. Conversely, exogenous S1P induces concentration-dependent intracellular calcium elevation and platelet aggregation via RhoA activation [68]. From the therapeutic perspective, platelet-rich plasma (PRP) therapy represents a promising strategy for diabetic foot ulcers (DFU) due to its pro-angiogenic, anti-inflammatory and low immunogenicity properties. PRP-derived exosomes (PRP-Exos) are enriched with S1P, which orchestrates angiogenesis during diabetic wound healing by activating the S1PR1/AKT/FN1 signaling pathway. Collectively, these findings provide a preliminary theoretical foundation for the clinical translation of PRP-Exos in future DFU therapies [69].

Multiple natural bioactive compounds also exert metabolic protective effects via sphingolipid modulation. Epigallocatechin-3-gallate (EGCG) synergizes with caloric restriction to modulate gut microbiota diversity, reduce plasma sphingolipid levels and alleviate insulin resistance, hepatic steatosis and obesity [70]. Chrysanthemum morifolium leaf extract and luteolin supplementation downregulate the expression of sphingolipid synthetic genes (Cers4, Cers6, Sgms1, Sgms2) in high-fat diet-fed mice, reducing lipotoxic ceramide and sphingomyelin accumulation to mitigate obesity related metabolic disorders. Traditional Chinese medicine Renshenjian (RSJ) reduced HIF-1a and SPTLC2 expression and lowered hepatic ceramide accumulation, contributing to attenuated dyslipidemia and liver damage in a mouse model of T2DM [71]. The interplay between fibroblast growth factors (FGFs) and sphingolipid metabolism has also garnered considerable attention. Fibroblast growth factor 21 (FGF21) remodels the profile of multiple ceramides and S1P in an adiponectin-independent manner to improve metabolic dysfunction [72]. Intriguingly, in the skeletal muscle of mice fed high-fat diets, *Cers1* expression is markedly upregulated, leading to robust accumulation of C18:0 N-acyl ceramide. This CERS1-derived C18:0 N-acyl ceramide pool drives obesity-associated insulin resistance by suppressing the secretion of the myokine FGF21 into the systemic circulation. Conversely, skeletal muscle-specific *Cers1* knockout mice exhibit elevated intramuscular and circulating FGF21 levels, which effectively ameliorate systemic glucose tolerance [73]. Additionally, fibroblast growth factor 13 (FGF13) acts as a key ceramide-dependent target of energy and glucose homeostasis. Ablation of FGF13 represents a promising therapeutic approach for obesity-associated insulin resistance and hepatic steatosis [74]. Moreover, serum levels of fibroblast growth factor 19 (FGF19), a hormone regulated by the farnesoid X receptor (FXR), are significantly increased in patients with hypercholesterolemia. *SMPD3*, an FXR target gene, positively correlates with circulating ceramide and cholesterol in the intestine. Thus, pharmacological inhibition of FXR using the antagonist glycoursodeoxycholic acid (GUDCA), intestine-specific genetic ablation of FXR or pharmacological inhibition of SMPD3 using GW4869 effectively attenuated atherosclerosis by reducing circulating ceramide levels [75].

**Figure 3:**
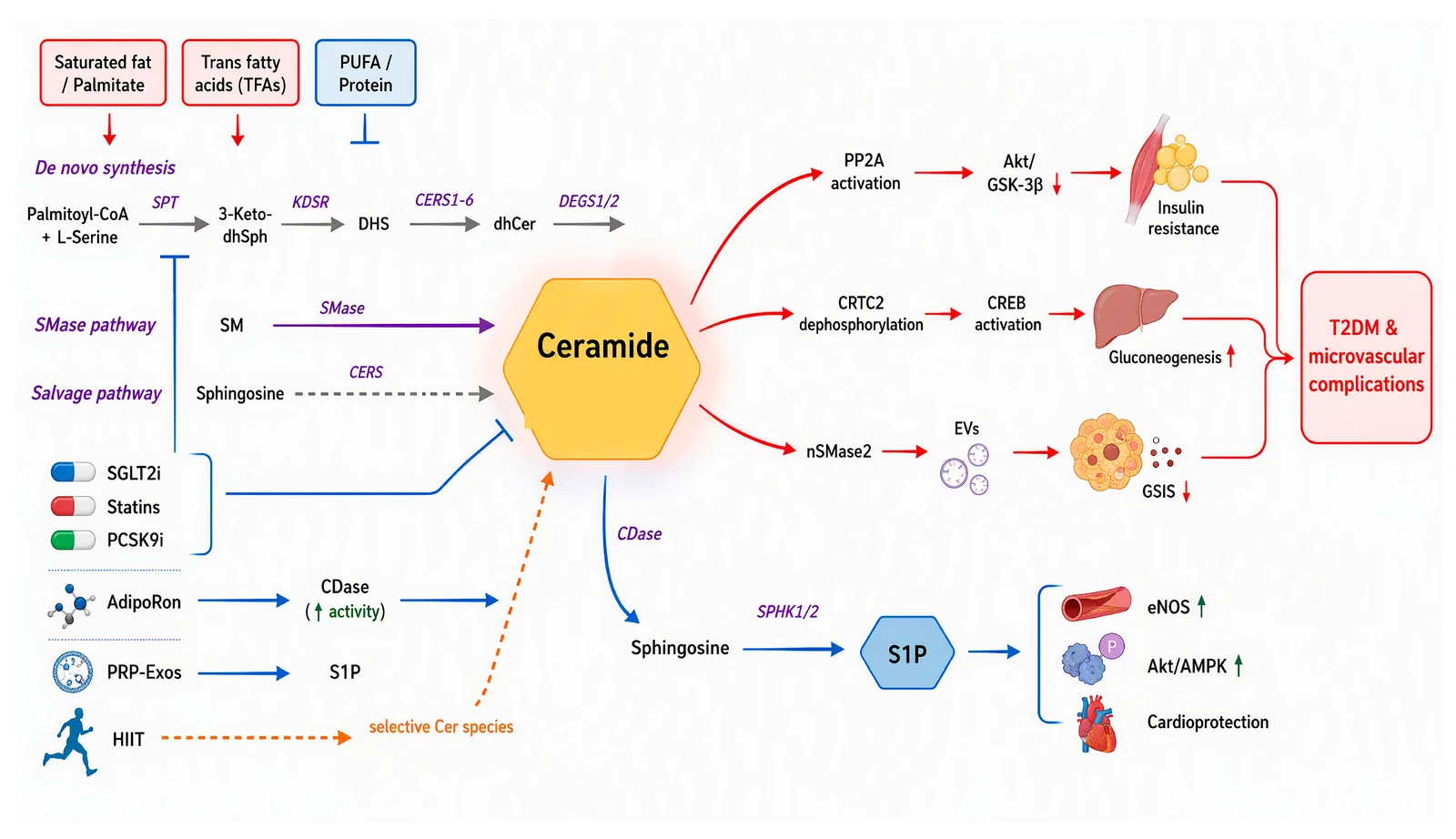
Schematic overview of sphingolipid metabolism and its dysregulation in T2DM. In T2DM, ceramide accumulation drives insulin resistance, hepatic gluconeogenesis and impaired GSIS through CRTC2/CREB activation and suppression of AKT/AMPK signaling. Conversely, conversion of ceramide to sphingosine-1-phosphate (S1P) by CDase and SPHK1/2 exerts cardioprotective effects via AKT/AMPK/eNOS activation. Therapeutic interventions including SGLT2i, statins, PCSK9 inhibitors, AdipoRon, HIIT, and PRP-Exos restore sphingolipid homeostasis, offering potential strategies to improve glycemic control and delay the progression of its complications. (Red = pathogenic/disease-driving; blue = protective/therapeutic; purple = SMase pathway; grey dashed = salvage pathway; orange dashed = HIIT-selective ceramide species. Solid arrows = conversion/activation/promotion; T-bars = inhibition; dashed arrows = indirect/alternative effects.

Taken together, these preclinical and early clinical findings suggest that these compounds may represent promising therapeutic strategies. However, most evidence comes from cell and animal models, and clinical translation will require large prospective trials with long-term safety evaluation. Importantly, tissue- and chain-length-specific modulation rather than global suppression of sphingolipid synthesis may offer a more precise approach. Nevertheless, clinical translation will demand robust evidence from large-scale prospective trials together with evaluations of long-term safety and off-target effects (Figure 3).

## 9. Discussion

Sphingolipids are crucial in T2DM, particularly in its CVD and microvascular complications. The sphingolipid rheostat has attracted increasing attention. It is mainly determined by the balance between ceramide and S1P, often assessed as their ratio. The accumulation of ceramide inhibits the signaling of AKT and promotes hepatic gluconeogenesis, and is positively correlated with glucolipotoxicity. S1P has been shown to enhance insulin sensitivity through the AKT and AMPK signaling pathways [76]. Ceramide accumulation drives pro-death autophagy, whereas S1P promotes protective autophagy. Through distinct downstream signaling pathways, they achieve opposite functional outputs and form the core mechanism by which the sphingolipid rheostat governs cell fate [77]. However, these sphingolipids and their related metabolic enzymes exert double-edged effects. Acute ceramide accumulation promotes its conversion to S1P, which activates eNOS to generate NO, causing vasodilation, whereas chronic ceramide accumulation induces vascular dysfunction and vasoconstriction [78]. Elevated plasma S1P is positively associated with an increased risk of hypertension and cardiovascular events. S1P/S1PR2 may mediate endothelial dysfunction by inhibiting the PI3K/AKT signaling pathway. Blockade of S1PR2 with the specific antagonist or small interfering RNA leads to increased NO levels, thereby ameliorating endothelial dysfunction [79,80]. Similarly, mitochondrial oxidative stress in renal tubular epithelial cells has been shown to promote S1P deposition in DN, along with elevated SPHK1 activity. Moreover, this abnormal S1P accumulation correlates closely with upregulated cPLA2 expression and ectopic lipid droplet formation [81]. ASM is a key enzyme in sphingolipid rheostat regulation through its catalysis of sphingomyelin hydrolysis to ceramide. Before extensive lysosomal lipid accumulation, ASM deficiency biases the rheostat toward S1P accumulation, thereby suppressing apoptosis. Conversely, under persistent LPS-induced inflammatory damage, sphingolipid reprogramming becomes uncontrolled, leading to unexpectedly pro-inflammatory responses. As seen in Niemann-Pick disease with congenital ASM deficiency [82,83]. Likewise, in macrophages, *Asm* ablation only partially rescues cells from apoptosis, because de novo ceramide synthesis can compensate and still execute apoptotic cell death [84]. These findings indicate that dysregulation of the sphingolipid rheostat is not limited to the ceramide/S1P ratio but involves the entire metabolic network. Under injury, sphingolipid metabolic overload and failure of compensatoryresponses may occur, which paradoxically exacerbates apoptosis.

Ceramide can insert into lipid rafts of the plasma membrane, the mitochondrial outer membrane, and the ER phospholipid bilayer, where it promotes inflammatory cascades, oxidative stress, and apoptotic signaling. However, the local ceramide concentrations in these membrane compartments and the downstream effectors of its pro-apoptotic pathway remain largely elusive. Lipid raft domains serve as platforms for the assembly of insulin signaling complexes. In T2DM, lipid overload and abnormal sphingomyelin metabolism disrupt the membrane environment of lipid rafts, leading to physical uncoupling of the insulin receptor from its substrates and downstream kinases [85,86,87]. In other words, total sphingolipid levels alone are of limited value. Crucial determinants include the subcellular sites of accumulation, the specific binding protein partners, and the effects on membrane organization and organelle crosstalk. This problem is further complicated by the multiple routes for ceramide and sphingomyelin synthesis and degradation. Some pathways, such as de novo synthesis, SM hydrolysis, and the salvage pathway, may dominate in specific tissues or disease stages, and their relative fluxes and kinetics remain incompletely defined. Sphingolipid species also differ in N-acyl chain length, saturation, and double-bond position. The ceramide synthase family CerS1-6 primarily determines chain length, but how these enzymes are balanced, how their expression varies across tissues, and whether they cooperate or compete in diabetes remain open questions. Addressing these issues should facilitate the development of more selective CerS inhibitors and organ-targeted therapies. Given that sphingolipid metabolism is compartmentalized across multiple organelles, organelle-level targeting may offer greater therapeutic precision than systemic inhibition.

Overnutrition and Western-style diets rich in saturated fatty acids and industrial trans fats drive ceramide accumulation that promotes insulin resistance, pancreatic β-cell dysfunction, and cardiovascular and microvascular complications. Conversely, diets enriched in unsaturated fatty acids, caloric restriction, and protein-rich regimens effectively restore sphingolipid homeostasis, underscoring the profound impact of dietary modulation [88]. Although supplementation with appropriate doses of sphingolipids may inhibit cholesterol absorption and improve the lipid profile, its absorption, transportat and metabolism in intestinal epithelial cells, as well as its effects on other gut microbiota, remain to be explored. HDL-associated apolipoprotein M-bound S1P exerts anti-inflammatory and vasoprotective effects, indicating that the carrier context can determine sphingolipid function. Exosomes are another underappreciated compartment. They are enriched in ceramide, S1P, and sphingosylphosphorylcholine and may deliver these lipids to recipient cells to modulate metabolic signaling. However, the sorting, release, and sensing of exosomal sphingolipids by target cells remain poorly understood. Moreover, sphingolipids have been implicated in epigenetic regulation and may contribute to metabolic memory or intergenerational transmission of metabolic traits, though the chromatin-level mechanisms are still largely unresolved.

This review has several limitations. First, it is a narrative review based on a non-systematic PubMed search; therefore, it may not capture all relevant studies and is susceptible to selection and publication bias. Second, no formal risk-of-bias assessment or meta-analysis was performed. Third, the evidence summarized here includes human observational studies, animal models, and in vitro experiments, which differ substantially in translational weight. In addition, because only PubMed was searched, database-related selection bias cannot be excluded. Most biomarker evidence summarized in this review derives from observational studies. Few circulating sphingolipid species have been validated in independent prospective cohorts using standardized analytical platforms, and even fewer have demonstrated incremental value beyond established clinical risk factors. Therefore, sphingolipids should currently be regarded as candidate biomarkers rather than established diagnostic, prognostic, or risk-prediction tools.

## 10. Conclusions

Sphingolipids are increasingly recognized as important mediators of T2DM. Conventional antidiabetic and lipid-lowering agents can influence sphingolipid metabolism in humans. In contrast, newer strategies such as blocking SPT/DEGS1 or activating ceramidase have shown early promise mainly in preclinical models, but clinical evidence in patients is still limited. Translating these discoveries into clinical practice will require progress in these areas. First, we need standardized analytical platforms, supported by large prospective cohorts, to establish how circulating and target tissue levels of ceramide/S1P and other sphingolipids differ between diabetic and non-diabetic individuals and whether they correlate with diabetic complications. These studies should also explore whether ceramides with different acyl-chain lengths are tied to specific disease features. Such data may eventually support earlier risk assessment and disease monitoring, if validated in prospective clinical studies. Second, nanoliposome-delivered siRNA against *SPT/DEGS1*, along with ceramidase activators and chain-length-selective modulators of CerS, must be tested in well-controlled human trials with extended safety follow-up. Third, beyond pharmacotherapy, integrating lifestyle interventions is essential. Dietary counseling, structured exercise, and evidence-based pharmacotherapy may yield additive or even synergistic benefits. Finally, it requires sustained cross-disciplinary collaboration and durable translational investment are required to converte these advances into tangible benefits to patients with T2DM.

## Data Availability

No new data were created or analyzed in this study. Data sharing is not applicable to this article.

## Abbreviations

S1P	Sphingosine-1-Phosphate
T2DM	Type 2 Diabetes Mellitus
DN	Diabetic Nephropathy
DR	Diabetic Retinopathy
CVD	Cardiovascular Diseases
NAFLD	Nonalcoholic Fatty Liver Disease
SPT	Serine Palmitoyltransferase
KDSR	3-Ketodihydrosphingosine Reductase
DHS	Dihydrosphingosine
CERS1-6	Ceramide Synthases 1-6
dhCer	Dihydroceramide
DEGS1/2	Sphingolipid Δ4-Desaturase 1/2
SMase	Sphingomyelinase
SM	Sphingomyelin
SMPD1	Sphingomyelin Phosphodiesterase 1
ASM	Acid Sphingomyelinase
nSMA	Neutral Sphingomyelinase
alk-SMA	Alkaline Sphingomyelinase
CDase	Ceramidase
SPH	Sphingosine
FFAs	Free Fatty Acids
SPHK1/2	Sphingosine Kinase 1/2
UGCG	UDP-glucose Ceramide Glucosyltransferase
SMS	Sphingomyelin Synthase
AKT	Protein Kinase B
AMPK	AMP-activated Protein Kinase
mTOR	Mammalian Target of Rapamycin
PP2A	Protein Phosphatase 2A
GSK-3β	Glycogen Synthase Kinase-3 beta
CREB	cAMP-responsive Element-binding Protein
CRTC2	CREB-regulated Transcription Coactivator 2
G6Pase	Glucose-6-Phosphatase
PEPCK	Phosphoenolpyruvate Carboxykinase
IRβ	Insulin Receptor beta
sXBP1	spliced X-box Binding Protein 1
GLUT2	Glucose Transporter 2
GLUT4	Glucose Transporter 4
IRS-1	Insulin Receptor Substrate-1
EVs	Extracellular Vesicles
GSIS	Glucose-stimulated Insulin Secretion
LPS	Lipopolysaccharide
SPBP	Sphingoid Base Phosphate
IR	Insulin Resistance
TFAs	Trans Fatty Acids
VLDL	Very Low-Density Lipoprotein
MFGMs	Milk Fat Globule Membranes
PS	Phytosphingosine
BMI	Body Mass Index
LDL-C	Low-Density Lipoprotein Cholesterol
eGFR	Estimated Glomerular Filtration Rate
mtDNA	Mitochondrial DNA
cGAS-STING	Cyclic GMP-AMP Synthase-Stimulator of Interferon Genes
C1P	Ceramide-1-Phosphate
NO	Nitric Oxide
eNOS	Endothelial Nitric Oxide Synthase
TNF-α	Tumor Necrosis Factor-alpha
NPDR	Non-Proliferative Diabetic Retinopathy
ACER2	Alkaline Ceramidase 2
FATP2	Fatty Acid Transport Protein 2
ER	Endoplasmic Reticulum
LacCer	Lactosylceramide
dhCer	Dihydroceramide
GDM	Gestational Diabetes Mellitus
SGLT2i	Sodium-glucose Cotransporter 2 Inhibitors
LPC	Lysophosphatidylcholine
PC	Phosphatidylcholine
PE	Phosphatidylethanolamine
TG	Triacylglycerol
PDR	Proliferative Diabetic Retinopathy
PCSK9	Proprotein Convertase Subtilisin/Kexin type 9
LNP	Lipid Nanoparticle
SREBP-1c	Sterol Regulatory Element-Binding Protein 1c
HFD	High-Fat Diet
DFU	Diabetic Foot Ulcers
PRP	Platelet-Rich Plasma
PRP-Exos	PRP-derived Exosomes
S1PR1	S1P Receptor 1
EGCG	Epigallocatechin-3-gallate
FGFs	Fibroblast Growth Factors
FGF21	Fibroblast Growth Factor 21
FGF13	Fibroblast Growth Factor 13
FGF19	Fibroblast Growth Factor 19
FXR	Farnesoid X Receptor
GUDCA	Glycoursodeoxycholic Acid
DM	Diabetes Mellitus
IL-11/IL-11Rα	Interleukin-11/Interleukin-11 Receptor alpha
GlcCer	Glucosylceramide
S1PR2	S1P Receptor 2
HDL	High-Density Lipoprotein
